# Single-stranded DNA binding protein 2 expression is associated with patient survival in hepatocellular carcinoma

**DOI:** 10.1186/s12885-018-5158-z

**Published:** 2018-12-12

**Authors:** Hyunsung Kim, Yeseul Kim, Yumin Chung, Rehman Abdul, Jongmin Sim, Hyein Ahn, Su-Jin Shin, Seung Sam Paik, Han Joon Kim, Kiseok Jang, Dongho Choi

**Affiliations:** 1 Departments of Pathology, College of Medicine, Hanyang University, 222 Wangsimni-ro, Seongdong-gu, Seoul 04763 South Korea; 2 Departments of Surgery, College of Medicine, Hanyang University222 Wangsimni-ro, Seongdong-gu, Seoul 04763 South Korea

**Keywords:** Hepatocellular carcinoma, SSBP2, Single-stranded DNA binding protein 2, Immunohistochemistry, Prognosis

## Abstract

**Background:**

SSBP2, single-stranded DNA binding protein 2, is a subunit of the ssDNA-binding complex that is involved in the maintenance of genome stability. The majority of previous studies have suggested a tumor-suppressive role of SSBP2, which is silenced by promoter hypermethylation in several human malignancies, such as hematologic malignancies, prostate cancer, esophageal squamous cell carcinoma, ovarian cancer, and gallbladder cancer. However, an oncogenic role of SSBP2 has been suggested in glioblastoma patients. We investigated the clinicopathologic significance of SSBP2 expression in hepatocellular carcinoma.

**Methods:**

We constructed tissue microarrays consisting of 21 normal liver parenchyma and 213 hepatocellular carcinoma tissues with corresponding adjacent non-neoplastic tissues. SSBP2 expression was investigated by immunohistochemistry, and positive expression was defined as more than 10% of the tumor cells to show nuclear staining. We then analyzed the correlations between SSBP2 expression and various clinicopathologic characteristics, and further studied the role of SSBP2 in cell growth and migration.

**Results:**

Hepatocytes were negative for SSBP2 immunohistochemistry in all normal liver samples, whereas the nuclei of normal bile duct epithelium and sinusoidal endothelium were immunoreactive. Positive immunoreactivity was found in one (0.6%) out of 180 non-neoplastic liver tissue samples adjacent to the tumor and in 16 (8.5%) out of 189 hepatocellular carcinomas. Positive SSBP2 expression was significantly correlated with tumor multifocality (*P* = 0.027, chi-square test), high histologic grade (*P* = 0.003, chi-square test), and frequent vascular invasion (*P* = 0.001, chi-square test). Kaplan-Meier survival curves revealed that patients with SSBP2 expression had poor prognosis in both disease-free and overall survival (*P* = 0.004 and *P* = 0.026, respectively, log-rank test). SSBP2-positive tumors also had a higher Ki-67 proliferation index (*P* <  0.001, t-test). Furthermore, downregulation of SSBP2 in the Huh7 cell line inhibited cell migration (*P* = 0.022, t-test) with altered expression of epithelial-mesenchymal transition markers.

**Conclusions:**

The minority of hepatocellular carcinomas expressed SSBP2 by immunohistochemistry, whereas normal hepatocytes were negative. SSBP2-positive hepatocellular carcinomas were significantly associated with aggressive phenotypes and poor clinical outcome.

## Background

Hepatocellular carcinoma (HCC) represents approximately 4% of newly diagnosed cancers worldwide. Approximately 560,000 people are diagnosed with HCC annually, and HCC is considered to show a poor prognosis [1]. In Korea, HCC is the second most common cause of cancer-related death among men and the third among women [2]. Therefore, it is important to discover novel therapeutic targets and prognostic biomarkers for hepatocellular carcinoma.

The human single-stranded DNA binding protein 2 (SSBP2) gene was identified as a candidate tumor suppressor of myeloid leukemia from a critical region of loss in chromosome 5q14.1 [3]. SSBP2 is a subunit of the ssDNA-binding complex that is involved in the maintenance of genome stability. SSBP2 binds the transcriptional adaptor protein Lim domain-binding protein 1 (LDB1) through a highly conserved N-terminal domain; consequently, LDB1 binds the LIM domains of LIM only proteins (LMO) or LIM homeodomain proteins (LHX). Although the precise levels of LMO, LHX, and LIM-binding proteins are critical for a number of developmental programs, accumulating evidence suggests the function of altered stoichiometry of these complexes during oncogenesis of various malignancies [3, 4].

The role of SSBP2 in solid organ tumors is still under investigation, with the results of previous studies being inconsistent as to whether SSBP2 functions as a tumor promoter or suppressor [5]. Most studies have suggested a tumor suppressive role of SSBP2, which is silenced by promoter hypermethylation in several human tumors, including hematologic malignancies, prostate cancer, esophageal squamous cell carcinoma, ovarian cancer, and gallbladder cancer [5–9]. However, an oncogenic role of SSBP2 has been suggested in glioblastoma patients [10].

Gene promoter methylations have been described at all stages that encompass carcinogenesis of HCC and have been considered as potential molecular markers for tumor initiation and progression [11]. Many studies focusing on CpG islands in gene promoter regions have identified several hypermethylated genes such as RASSF1A, B4GALT1, and SSBP2 in HCCs [11]. However, the expression level of SSBP2 and its clinicopathologic significance in HCC remains unclear.

In this study, we investigated the immunohistochemical expression of SSBP2 in normal liver parenchyma and hepatocellular carcinoma tissues with corresponding adjacent non-neoplastic tissues. SSBP2 expression was compared with various clinicopathologic characteristics, the proliferation marker ki-67 labelling index, and patient survival. Moreover, we studied the functional role of SSBP2 in terms of cell proliferation and migration using an HCC-derived cell line.

## Methods

### Patients and tumor samples

We enrolled a consecutive series of 213 patients with HCC in this study. All patients were diagnosed and underwent surgery at Hanyang University Hospital (Seoul, Korea) between 1991 and 2013. Patients with no complete clinical follow-up data or available paraffin blocks were excluded, leaving 189 patients. The clinicopathologic characteristics of these cases are summarized in Table 1. The mean follow-up period was 57 months. All patients had received curative surgical resections with negative resection margins. No additional treatment was performed in 74 (39.1%) patients. Transarterial chemoembolization was performed in 90 (47.6%) patients, systemic chemotherapy in 8 (4.2%), radiofrequency ablation in 5 (2.6%), and transplantation in 1 (0.5%). According to the seventh edition of the American Joint Committee on Cancer (AJCC) staging system, 164 cases were Stage I or II and 25 were Stage III or IV. In addition, paired non-neoplastic liver tissue adjacent to the HCC, along with 21 normal liver tissue samples resected from patients with traumatic liver injury, were selected to compare SSBP2 expression. We reviewed all hematoxylin and eosin (H&E)-stained slides, pathology reports, and other medical records to confirm the diagnosis. The clinicopathologic parameters assessed were tumor size, tumor focality (number of tumor nodules, single or multiple), histologic grade (4-tier Edmondson-Steiner grading system), small vessel invasion (presence of tumor emboli within central hepatic vein, portal or large capsular vessel, microscopically), large vessel invasion (presence of tumor emboli in major branch of hepatic vein or portal vein, grossly as well as microscopically), AJCC stage, Ki-67 labeling index (eyeball estimation of TMA cores), serum alpha-fetoprotein (AFP), and patient survival. The pathological evaluation was performed by two pathologists (KJ and HK).

**Table 1:** Summary of clinicopathologic characteristics of hepatocellular carcinoma patients enrolled in this study (*n* = 189)

Clinicopathologic characteristics	Value (%)
Number of patients	189 (100%)
Mean age at surgery (years)	64.6 (±10.78)
Sex	
Male	148 (78.3%)
Female	41 (21.7%)
Etiology	
HBV	151 (79.9%)
HCV	11 (5.8%)
Alcohol	10 (5.3%)
Others	17 (9.0%)
Underlying liver disease	
Cirrhosis	165 (87.3%)
Hepatitis	18 (9.5%)
Others	6 (3.2%)
BLCL stages	
A	101 (53.4%)
B	9 (4.8%)
C	79 (41.8%)
Histologic grade	
Grade 1	12 (6.3%)
Grade 2	65 (34.4%)
Grade 3	93 (49.2%)
Grade 4	19 (10.1%)
AJCC stages	
I	95 (50.3%)
II	69 (36.5%)
III	5 (2.6%)
IV	20 (10.6%)
Child-Pugh class	
A	176 (93.1%)
B	13 (6.9%)
AFP^a^	
< 400	123 (65.1%)
≥ 400	40 (21.2%)
Surgical approach	
Wedge resection	41 (21.7%)
Partial hepatectomy	148 (78.3%)
Additional treatments	
None	74 (39.1%)
Systemic chemotherapy	8 (4.2%)
Radiofrequency ablation	5 (2.6%)
Transarterial chemoembolization	90 (47.6%)
Transplantation	1 (0.5%)
Combined (2 or 3 of above)	11 (5.9%)

^a^α-fetoprotein, 26 cases missed

### Tissue microarray construction

We used a manual tissue microarrayer (Unitma, Seoul, Korea) for tissue microarray construction from archival formalin-fixed, paraffin-embedded tissue blocks. The non-necrotic central portion of the carcinoma, spanning 0.5 cm or larger, was selected by light microscopy of H&E-stained sections. Tissue cylinders of 2-mm diameter were punched from a previously marked lesion on each donor block and transferred to the recipient block (Unitma). Each tissue microarray was comprised of 5 × 10 samples.

### Immunohistochemical staining

The immunohistochemical staining for SSBP2 was performed with 4-μm-thick sections from TMA blocks. The sections were deparaffinized in xylene and then rehydrated through graded ethanol. For antigen retrieval, we performed autoclave heating at 100 °C for 20 min in sodium citrate buffer (pH 6.0). Endogenous peroxidase activity was blocked with peroxidase blocking solution (S2023, Dako, Glostrup, Denmark). TMA slides were incubated with primary antibodies at 4 °C overnight and then incubated with labeled polymer (EnVision/HRP, K5007, Dako) for 30 min at room temperature. The primary rabbit monoclonal [EPR11520] antibody was raised against a synthetic peptide corresponding to the human SSBP2 (amino acid sequence 300 to the C-terminus) (ab177944, Abcam, Cambridge, MA). 3, 3′-Diaminobenzidine tetrahydrochloride was used as the chromogen for detection, and Mayer’s hematoxylin counterstain was applied. Immunostaining for Ki-67 was performed using the Bond-Max automated immunostainer (Leica Biosystems, Wetzlar, Germany) with anti-Ki-67 antibody (Dako, M7240).

### Interpretation of immunohistochemical staining

**Fig. 1:**
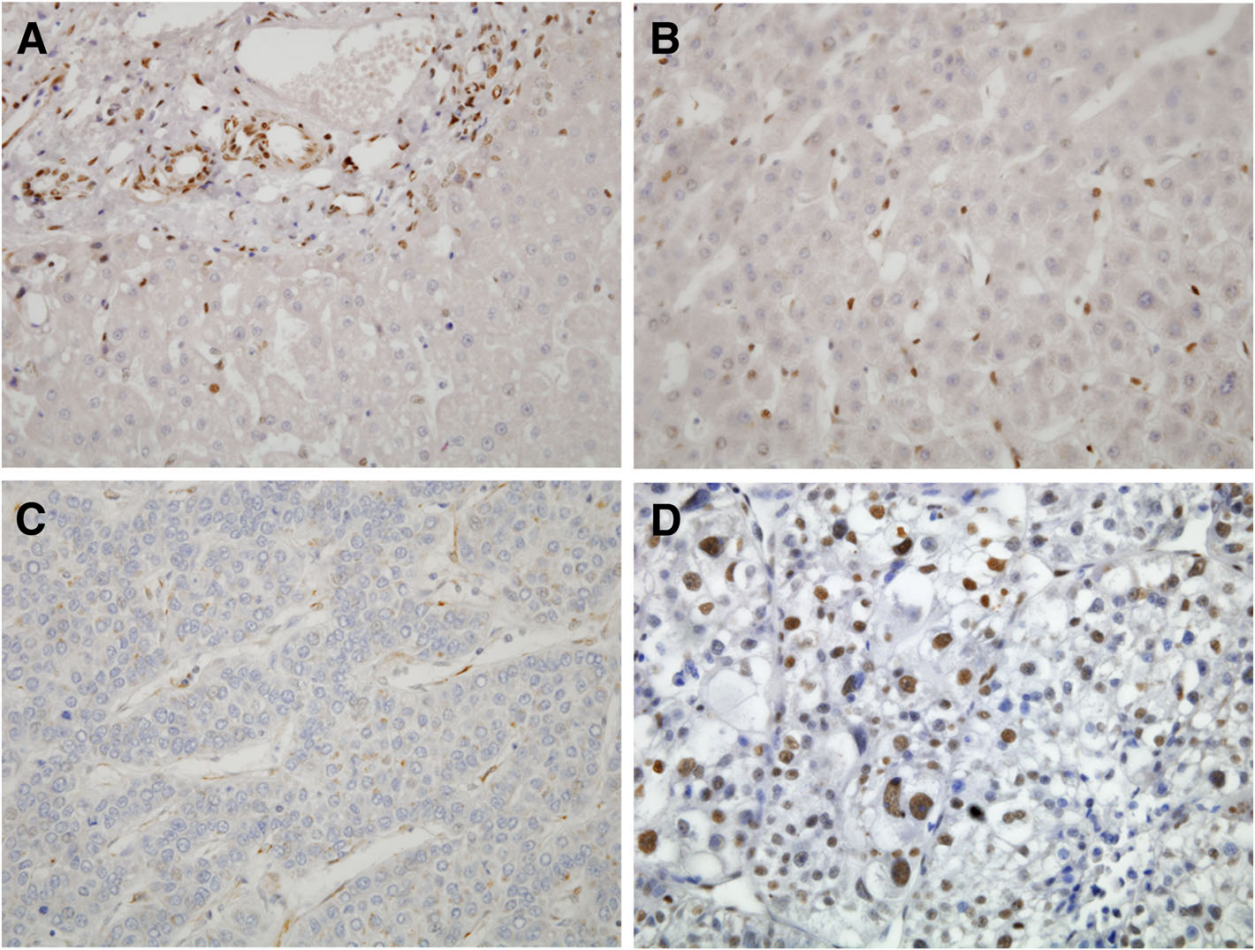
SSBP2 expression in human liver tissues. Representative sections of immunohistochemical staining for SSBP2 in normal liver tissue (**a**) (original magnification × 400), adjacent non-neoplastic liver tissue (**b**) (original magnification × 400), and hepatocellular carcinoma (**c**, **d**) (original magnification × 400). Whereas the nuclei of normal bile duct epithelium and sinusoidal endothelium are positive, the hepatocytes of normal and adjacent non-neoplastic liver tissues are all negative for SSBP2, and a subset of hepatocellular carcinomas shows positive staining. Representative HCC sample showing negative (**c**) and positive (**d**) nuclear staining in tumor cells

SSBP2 expression was evaluated semi-quantitatively by two independent pathologists (HK and KJ) who were blind to patient clinical outcome. Positive expression was defined as more than 10% of the tumor cells to show nuclear staining [12]. A representative microscopic image with positive staining in HCC is shown in Fig. 1.

### Cell lines and siRNAs

The human HCC cell line (Huh7) was purchased from the Korean Cell Line Bank (Seoul, Korea). Huh7 cells were cultured in RPMI supplemented with 10% FBS at 37 °C with a 5% CO2 concentration. The human siRNA SSBP2 (Bioneer, Daejeon, Korea) and negative control siRNA (Bioneer) were transfected with Lipofectamine 2000 (Invitrogen, Carlsbad, CA, USA) in a 6-well culture plate and harvested after 48 h.

### Cell proliferation assay

At 48 h after transfection, cells were digested using trypsin for 5 min and then seeded into six 6-well plates, followed by incubation for 5 days. Each plate was harvested daily. The viable cell numbers were manually obtained with a hemocytometer after trypan blue staining. The experiments were performed in triplicate.

### Transwell migration assay

The Transwell assay was performed with a Boyden chamber and 10^5^ transfected cells suspended in the upper chamber with serum free media, which was transferred to a 24-well lower chamber (RPMI containing 10% FBS). Then, 24 h after incubation at 37 °C, the upper cells were removed by wiping the surface of the membrane with cotton swabs. The migrated cells on the lower side were fixed with formalin for 10 min and stained with 1% crystal violet. In 10 microscopic fields (× 200), migrated cells were counted, and mean values were compared. The experiments were performed in triplicate.

### Western blot

Huh7 cell lines transfected with SSBP2 siRNA or NC-siRNA were harvested, and total cell lysates were prepared. The cell lysates were separated by SDS-PAGE and transferred onto nitrocellulose membranes. After blocking with 5% skim milk, the membranes were incubated with antibodies against SSBP2 (rabbit monoclonal, ab177944, Abcam), E-cadherin (rabbit monoclonal, #3195, Cell Signaling, Danvers, MA, USA), N-cadherin (rabbit monoclonal, #13116, Cell Signaling), TCF8/Zeb1 (rabbit monoclonal, #3396, Cell Signaling), Twist1 (rabbit monoclonal, #46702, Cell Signaling), and Beta-actin (rabbit monoclonal, #4970, Cell Signaling). The bound antibodies were detected with HRP-conjugated secondary antibodies and visualized using chemiluminescence reagent (Amersham Biosciences, Little Chalfont, UK).

### Statistical analysis

Statistical analysis was performed using SPSS software version 21 (IBM Corp., Armonk, USA). The chi-square test was used to evaluate the correlations between SSBP2 expression and clinicopathologic parameters of tumor size, histologic grade, vascular invasion, AJCC stage, and serum AFP. Disease-free survival and overall survival were determined using Kaplan-Meier survival curves, and the log-rank test was used to compare the differences. The Cox proportional hazard regression model was used to evaluate the independent prognostic significance. Differences in Ki-67 labeling index and experimental data were compared by Student’s t-test. *P* <  0.05 was considered as statistically significant.

## Results

### SSBP2 expression in human liver tissues

SSBP2 expression was evaluated in normal human liver tissue, non-neoplastic liver parenchyma adjacent to HCC, and HCC tissue (Fig. 1). Normal hepatocytes were negative for SSBP2 in all 21 normal liver tissue samples, whereas the nuclei of normal bile duct epithelium and sinusoidal endothelium were immunoreactive. Positive immunoreactivity was found in one (0.6%) out of 180 non-neoplastic liver parenchyma samples and in 16 (8.5%) out of 189 HCC tissue samples (Table 2). SSBP2 was more frequently expressed in HCC tumor tissues compared to normal liver or adjacent non-neoplastic tissues (*P* < 0.001, chi-square test for linear trend).

**Table 2:** SSBP2 expression in normal liver, adjacent non-neoplastic liver tissue and HCC

Liver tissue	*n*	Immunoreactivity of SSBP2		*P*-value*
		Negative	Positive	
Normal	21	21 (100%)	0 (0%)	< 0.001
Adjacent non-neoplastic	180	179 (99.4%)	1 (0.6%)	
HCC	189	173 (91.5%)	16 (8.5%)	

*Chi-square for linear trend

### Correlations between SSBP2 expression and clinicopathologic characteristics

Table 3 shows the correlations between SSBP2 expression and clinicopathologic parameters. Positive SSBP2 expression was significantly correlated with tumor multifocality (*P* = 0.027, chi-square test), high histologic grade (*P* = 0.003, chi-square test), and frequent vascular invasion (*P* = 0.001, chi-square test). There was no statistically significant correlation between SSBP2 expression and tumor size, AJCC stage, or serum AFP. These results demonstrated that SSBP2 expression correlated with an aggressive histologic phenotype of HCC.

**Table 3:** Correlation between SSBP2 expression and clinicopathologic features in hepatocellular carcinoma

Clinicopathologic features	*n*	SSBP2 expression		*P*-value (chi-square test)
		Negative (%) (*n* = 173)	Positive (%) (*n* = 16)	
Tumor size				0.895
< 5 cm	121	111 (91.7%)	10 (8.3%)	
≥ 5 cm	68	62 (91.2%)	6 (8.8%)	
Focality				0.027
Single	156	146 (93.6%)	10 (6.4%)	
Multiple	33	27 (81.8%)	6 (18.2%)	
Histologic grade				0.003
Grade 1 & 2	77	76 (98.7%)	1 (1.3%)	
Grade 3 & 4	112	97 (86.6%)	15 (13.4%)	
Small vessel invasion				0.001
Not identified	109	106 (97.2%)	3 (2.8%)	
Present	80	67 (83.8%)	13 (16.2%)	
Large vessel invasion				0.267
Not identified	169	156 (92.3%)	13 (7.7%)	
Present	20	17 (85.0%)	3 (15.0%)	
Perineural invasion				0.348
Not identified	184	169 (91.8%)	15 (8.2%)	
Present	5	4 (80.0%)	1 (20.0%)	
AJCC stage				0.146
I & II	164	152 (92.7%)	12 (7.3%)	
III & IV	25	21 (84.0%)	4 (16.0%)	
Ki-67 index				
Mean (%)	189	3.93 (±9.80)	20.63 (±21.44)	< 0.001*

*Student t-test

### Correlations between positive SSBP2 expression and disease-free and overall survival

**Fig. 2:**
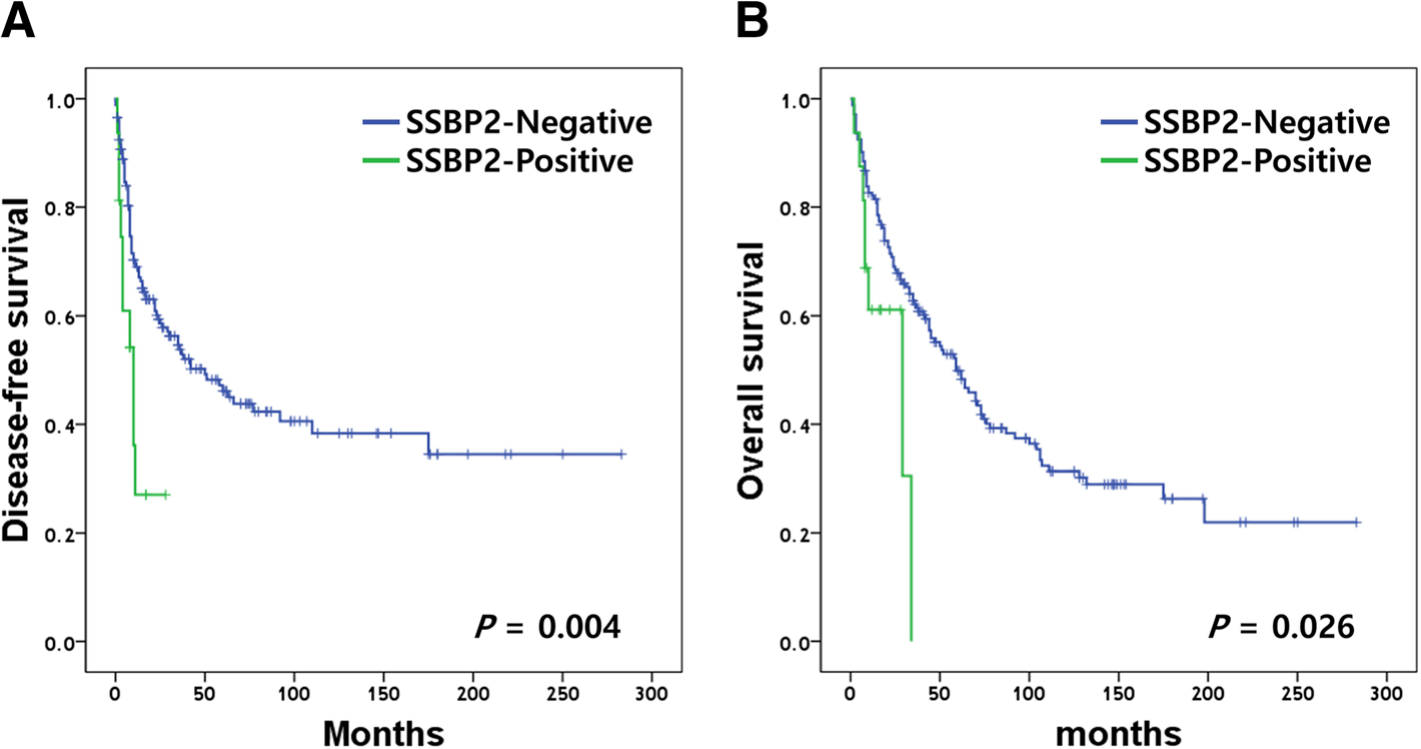
Kaplan-Meier survival curves of hepatocellular carcinoma patients stratified by SSBP2 expression. **a** Disease-free survival (log-rank test, *P* = 0.004), **b** Overall survival (log-rank test, *P =* 0.026)

The associations between conventional prognostic factors and patient survival were explored. Univariate survival analyses revealed histologic grade (*P* = 0.008), AJCC stage (*P* < 0.001) and vessel invasion (*P* < 0.001) as predictors of poor disease-free survival (DFS) in HCC patients (Table 4). Similarly, histologic grade (*P* = 0.005), primary tumor size (*P* = 0.002), AJCC stage (*P* < 0.001), and vessel invasion (*P* < 0.001) were negatively correlated with overall survival (OS) (Table 4). SSBP2 expression was demonstrated as a predictor of poor DFS and OS by univariate Cox regression analysis (*P* = 0.004 and *P* = 0.027, respectively). Kaplan-Meier survival curves revealed that patients with SSBP2-positive HCC had poor prognosis in both DFS and OS (*P* = 0.004 and *P* = 0.026, respectively, log-rank test) (Fig. 2). However, SSBP2 expression was not an independent prognostic factor for disease-free or overall survival in the multivariate analysis with the Cox proportional hazards model (*P* = 0.117 and *P* = 0.138, respectively) (Table 5). Subgroup analysis stratified by AJCC and BLCL stage failed to demonstrate the survival difference between SSBP2-positive and negative patients.

**Table 4:** Univariate Cox regression analysis of prognostic factors for disease-free survival and overall survival in hepatocellular carcinoma

Variables	Disease-free survival		Overall survival	
	HR (95% CI)	*P*-value	HR (95% CI)	*P-*value
SSBP2 expression (negative vs. positive)	2.652 (1.355–5.190)	0.004	2.323 (1.101–4.901)	0.027
Tumor size (< 5 cm vs. ≥ 5 cm)	1.104 (0.723–1.685)	0.648	1.792 (1.237–2.595)	0.002
Focality (single vs. multiple)	1.553 (0.956–2.523)	0.075	0.904 (0.552–1.481)	0.689
Histologic grade (grade 1&2 vs. 3&4)	1.763 (1.161–2.676)	0.008	1.744 (1.188–2.562)	0.005
Small vessel invasion	2.903 (1.932–4.362)	< 0.001	2.586 (1.783–3.750)	< 0.001
Large vessel invasion	3.486 (1.944–6.253)	< 0.001	3.165 (1.874–5.348)	< 0.001
Perineural invasion	2.067 (0.649–6.585)	0.219	1.920 (0.604–6.105)	0.269
AJCC staging (I & II vs. III & IV)	3.205 (1.871–5.491)	< 0.001	2.528 (1.536–4.162)	< 0.001

**Table 5:** Multivariate Cox regression analysis of prognostic factors for disease-free survival and overall survival in hepatocellular carcinoma

Variables	Disease-free survival		Overall survival	
	HR (95% CI)	*P-*value	HR (95% CI)	*P-*value
SSBP2 expression (negative vs. positive)	1.748 (0.869–3.517)	0.117	1.777 (0.831–3.803)	0.138
Histologic grade (grade 1&2 vs. 3&4)	1.645 (1.069–2.530)	0.023	1.692 (1.143–2.505)	0.009
AJCC staging (I & II vs. III & IV)	2.906 (1.671–5.051)	< 0.001	2.526 (1.529–4.173)	< 0.001

### Correlation between SSBP2 expression and tumor proliferation

SSBP2-positive tumors had a higher Ki-67 proliferation index than SSBP2-negative tumors (*P* < 0.001, t-test) in 189 clinical HCC samples (Table 3). To determine the role of SSBP2 in cell proliferation, we examined the effect of SSBP2 downregulation on HCC cell proliferation in vitro. The HCC cell line, Huh7, was transfected with siRNA specifically targeting SSBP2. The knockdown efficiency of SSBP2 in cell lines was confirmed by Western blot. However, there was no significant difference in cell proliferation between the negative control and the SSBP2-downregulated groups (data not shown).

### Downregulation of SSBP2 inhibited HCC cell migration and reversed EMT tendency

**Fig. 3:**
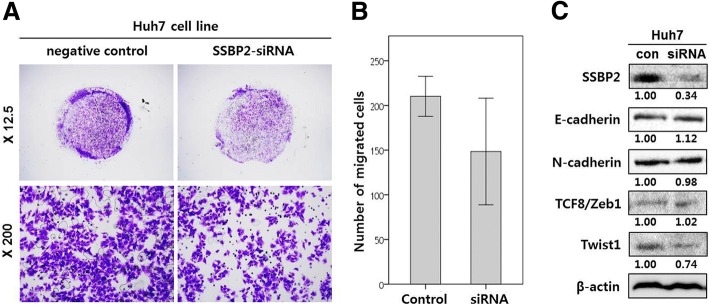
Downregulation of SSBP2 inhibits HCC cell migration. **a** Representative microscopic images of cells that migrated through the pores after 24 h in a transwell migration assay. **b** Migrated cells were counted, and the numbers were compared between the groups (*P* = 0.022, t-test). **c** Western blot and densitometric analyses for epithelial mesenchymal transition markers in Huh7 cell line following transfection with control and SSBP2 si-RNA

SSBP2 expression was correlated with vascular invasion in HCC patients (Table 3). To further evaluate the role of SSBP2 in tumor cell invasion, we performed a Transwell invasion assay in normal HCC cell lines and HCC cell lines (Huh7) transfected with SSBP2 siRNA. The knockdown efficiency of SSBP2 was confirmed by Western blot. SSBP2 downregulation restrained the migratory capacity of Huh7 cells (*P* = 0.022, t-test) (Fig. 3a and b). Western blots for EMT markers showed that SSBP2 downregulation increased the epithelial maker level (E-cadherin) and decreased the mesenchymal marker level (Twist1) (Fig. 3c). These results support the concept that SSBP2 promotes invasiveness in HCC.

## Discussion

Although SSBP2 is a well-known candidate tumor suppressor gene in acute myelogenous leukemia, the role of SSBP2 as a tumor promoter vs. a tumor suppressor in solid organ tumors is still controversial [5]. The majority of previous studies have suggested a tumor-suppressive role of SSBP2, which is silenced by promoter hypermethylation in most solid tumors, such as prostate cancer, esophageal squamous cell carcinoma, ovarian cancer, and gallbladder cancer. *Liu* et al. demonstrated that SSBP2 hypermethylation was observed in 54 out of 88 (61.4%) prostate cancer samples, but in none of the 23 (0%) benign prostatic hyperplasia samples. Furthermore, hypermethylation of SSBP2 was closely associated with higher stages, and forced expression of SSBP2 inhibited prostate cancer cell proliferation in a colony formation assay and caused cell cycle arrest [6]. *Huang* et al. showed that promoter methylation and downregulation of SSBP2 expression were frequently detected in esophageal squamous cell carcinoma samples and suggested a tumor suppressive role through inhibition of the Wnt signaling pathway [7]. *Brait* et al. analyzed promoter methylation of 13 genes including SSBP2 in ovarian cancer. The results revealed that hypermethylation of SSBP2 was observed in 3 out of 33 (9%) ovarian cancer samples; however, there was no statistical significance [11]. *Tsukamoto* et al. showed that promoter methylation of SSBP2 was observed in gallbladder cancer significantly more than in cholecystitis [9]. However, an oncogenic role of SSBP2 has been suggested in glioblastoma patients. *Xiao* et al. investigated transcript levels of SSBP2 in 619 glioblastoma patients. Increased expression of SSBP2 was significantly associated with poor overall survival among glioblastoma patients [10].

There are few studies concerning the function of SSBP2 in HCC. *Christina* et al. used a genome-wide epigenomic platform to discriminate HCC from non-HCC tissue [11]. Three genes, SSBP2, RASSF1A, and B4GALT1, were selected for a panel of methylation phenotypes. Most HCCs (78%) showed methylation of more than one gene, while about half of the adjacent normal samples (44%) showed methylation of one gene. SSBP2 was methylated in 14 (52%) out of 27 HCC samples and 6 (33%) out of 18 adjacent normal liver samples. Although methylation is a common epigenetic signaling tool that leads to gene silencing, various other mechanisms are involved in the regulation of gene expression [13]. The study conducted by *Christina* et al. only confirmed methylation of SSBP2 in HCC, but the actual expression level of SSBP2 in HCC has not yet been determined. We investigated the actual protein expression of SSBP2 in HCC, and correlated positive expression and clinicopathologic parameters. SSBP2 positivity was more frequently observed in cases with tumor multifocality, higher histologic grade, and vascular invasion. These results suggest that SSBP2 expression is closely associated with aggressive clinical behavior in HCC. In the survival analyses, the Kaplan-Meier survival curves revealed that there were significant differences between SSBP2-positive and -negative groups in both disease-free and overall survival. These results suggest that SSBP2 can be a poor prognostic factor in HCC.

Ki-67 protein is a nuclear protein associated with somatic cell proliferation [14]. The Ki-67 proliferation index is a well-known biomarker for poor prognosis and clinical deterioration in HCC [15]. In this study, there was a significant correlation between SSBP2 expression and high Ki-67 labelling index. However, an in vitro cell proliferation study using the Huh7 cell line failed to demonstrate the difference between control and SSBP2-downregulated cells. Although there are several studies describing SSBP2 as a regulator of cell cycle by C-MYC regulation in myelomonocytic cells, there is not yet a confirmative study disclosing the specific molecular pathway and function of the SSBP2 protein in tumor cell proliferation in solid organs [5]. Further investigation is needed to clarify the role of SSBP2 in tumor proliferation.

The correlation between SSBP2 expression and vascular invasion suggests a pivotal role of SSBP2 in the metastatic tendency of HCC. In vitro, downregulation of SSBP2 expression in the Huh7 cell line inhibited cell migration in the Transwell assay and promoted an increase in E-cadherin and a decrease in Twist1. The tendency to reverse EMT indicates that SSBP2 may play an important role in tumor invasion and distant metastasis. However, these results are not conclusive, due to the retrospective analysis of the clinical sample with potential bias, and the use of one HCC cell line.

Although our study revealed an oncogenic role of SSBP2 in HCC, there are some limitations. We used a retrospective study design, which has possibilities of bias and needs larger number of samples. Treatment and underlying disease have a great impact on the prognosis of HCC, however, subgroup analyses revealed no statistical significance due to a low SSBP2 positive rate and a small sample size. Concerning in vitro studies, we used only one HCC cell line (Huh7) for functional assay, and Western blot analysis for EMT markers showed a subtle difference between control and siRNA knockdown groups. Since we had no data on SSBP2 genetic alteration matched with immunohistochemical staining results, we could not identify the specific mutation of SSBP2 or molecular pathway in which SSBP2 acts as an oncogenic gene. To date, no study has reported specific mutations in SSBP2, and only The Cancer Genome Atlas data has revealed SSBP2 amplification in 0.5% of HCC patients (http://www.cbioportal.org). Further investigation is needed to clarify the cancer-specific mechanism of SSBP2.

In conclusion, we evaluated the clinicopathologic significance of SSBP2 expression in 189 HCC patients. The minority of HCCs expressed SSBP2 by immunohistochemistry, whereas normal hepatocytes were negative. SSBP2-positive HCCs were significantly associated with aggressive phenotypes and poor clinical outcome.

## Abbreviations

AFP: Alpha-fetoprotein
AJCC: the American Joint Committee on Cancer
DFS: Disease-free survival
H&E: Hematoxylin and eosin
HCC: Hepatocellular carcinoma
LDB1: Lim domain-binding protein 1
LHX: LIM homeodomain proteins
LMO: LIM only proteins
OS: Overall survival
SSBP2: Single-stranded DNA binding protein 2
